# Improvement of Processing Speed in Executive Function Immediately following an Increase in Cardiovascular Activity

**DOI:** 10.1155/2013/212767

**Published:** 2013-09-25

**Authors:** Nicoladie D. Tam

**Affiliations:** Department of Biological Sciences, University of North Texas, Denton, TX 76023, USA

## Abstract

This study aims to identify the acute effects of physical exercise on specific cognitive functions immediately following an increase in cardiovascular activity. Stair-climbing exercise is used to increase the cardiovascular output of human subjects. The color-naming Stroop Test was used to identify the cognitive improvements in executive function with respect to processing speed and error rate. The study compared the Stroop results before and immediately after exercise and before and after nonexercise, as a control. The results show that there is a significant increase in processing speed and a reduction in errors immediately after less than 30 min of aerobic exercise. The improvements are greater for the incongruent than for the congruent color tests. This suggests that physical exercise induces a better performance in a task that requires resolving conflict (or interference) than a task that does not. There is no significant improvement for the nonexercise control trials. This demonstrates that an increase in cardiovascular activity has significant acute effects on improving the executive function that requires conflict resolution (for the incongruent color tests) immediately following aerobic exercise more than similar executive functions that do not require conflict resolution or involve the attention-inhibition process (for the congruent color tests).

## 1. Introduction

Physical exercise is known to improve brain functions based on a large body of evidence, ranging from the improvement in academic performance in children to the improvement in cognitive function in both healthy subjects and patients with mental disorders. Numerous studies have shown that exercise is linked to an improvement in academic achievement for schoolchildren [1–5]. The improvement in academic performance is greater for physically fit children than for obese children [6]. Exercise has shown to improve brain processing speed by decreasing the event-related brain potential P300 in healthy subjects with sedentary lifestyles [7]. Brain imaging studies also showed that exercise improves cognitive functions by altering the efficiency of the neural circuitry based on functional magnetic imaging (fMRI) data, especially for overweight children [8].

Physical activities can improve memory and cognitive impairment in Alzheimer's patients and motor functions in Parkinson's patients. It improves the logical memory and reduces the whole brain cortical atrophy in older adults with mild cognitive impairment (MCI) [9]. Treadmill exercise has been shown to enhance memory plasticity function in hippocampus through (brain-derived neurotrophic factors BDNF) upregulation (in rats), which is potentiated by antidepressant treatment [10]. It reverses stress-induced changes by establishing functional reconnection of hippocampal synapses that is mediated by antidepressant actions [11]. These changes have been shown to be related to gene expression in the hippocampus resulted from treadmill exercise [12]. Exercise can improve motor functions in Parkinson's patients by enhancing motor circuitry connectivity based on the functional connectivity MRI (fcMRI) brain imaging studies [13]. These long-term effects of exercise on brain functions are well established in both children and adults, in physically fit and overweight individuals, in both healthy subjects and patients, and in individual trials and randomized-control trials [14].

In this study, we will focus on immediate effects of exercise on executive function, instead of long-term effects. Executive control is one of the essential cognitive processes in higher animals to assess and evaluate information, so they can make an appropriate decision in response to the stimuli. It is the higher-level cognitive function that requires selective attention, judgment, anticipation, planning, and reasoning to perform the crucial decision-making functions [15]. But when conflicts arise, an appropriate decision is needed to choose one out of many options in order to resolve such conflicts. Conflicts arise when there are multiple competing options to choose from, but only one of the options can be selected. That is, a person must choose one but not both at the same time. Choosing one option effectively conflicts with the other options, resulting in nullifying the competing options—that is, the dilemma created by “wanting to have the cake, and eat it too.” If the conflict is not resolved, it will result in a deadlock, thus preventing a decision from being made, when the desire is to want both, but it is impossible to have both. Thus, unresolved conflict interferes with the decision-making process. To resolve a conflict, it requires an attention-inhibition process to break the deadlock. It is resolved by suppressing one of the choices, in order to select the alternative option to make a decision. Insufficient inhibition often leads to impulsivity, by choosing the inappropriate action instead of the appropriate one. Impulsivity arises when the inappropriate action cannot be overridden by the appropriate one, due to a lack of inhibition to the inappropriate choice.

Numerous studies have shown that long-term exercise programs can improve the executive function in school-aged children [16]. These improvements are related to an increased bilateral prefrontal cortex activity and a reduced bilateral posterior parietal cortex activity based on fMRI studies [17]. Exercise can improve reaction time, without compromising accuracy [18]. Acute exercise improves executive function in (attention deficit hyperactivity disorder ADHD) children, using Wisconsin Card Sorting Test (WCST), regardless of age [19], while the associated increase in the middle cerebral artery blood flow velocity (MCAv) is related to cognition at rest (not during exercise). The relationship between different hemodynamics measures becomes uncoupled during exercise in terms of global cerebral blood flow, regional cortical oxygenation, and cortical hemodynamics blood volume [20].

Toward the goal to determine the immediate effects of exercise on brain function, we will design an experiment to identify the cognitive improvements immediately after physical activities. One of the classic psychophysics tests is the Stroop Test [21–23] that addresses this executive function by invoking an attention-inhibition process to resolve the conflicting interference. It is a simple name-that-color test that requires a human subject to name the color of a word, instead of reading the color name. When the word (color name) matches the color in which the word is printed on, such as “RED,” no conflict occurs. But when the word (color name) does not match the color in which the word is printed on, such as “RED,” a conflict is elicited, because the subject has to choose whether to name the color (blue) or read the color name (red), but not both. This causes the classic interference in the executive control, when the color-naming process conflicts with the word-recognition process in the brain. In order to name the color, the word-recognition process has to be suppressed; otherwise, the word-recognition will supersede the color recognition, resulting in naming the wrong color. It is one of the many classic cognitive tests for identifying the impulsivity of the subjects that requires executive control by the prefrontal cortex [15]. Using the example illustrated above in the naming of the color of the word “RED,” the subject has to suppress what the word says (which says red), in order to identify the color as “blue” not red. The tendency is to read the word as “red” impulsively rather than saying it is “blue.” In order to resolve this color conflict, the task requires the attention-inhibition process in the prefrontal cortex to suppress the word-recognition process, so that the color-recognition process can supersede the impulsivity to read the word instead of identifying the color.

## 2. Methods

**Figure 1:**
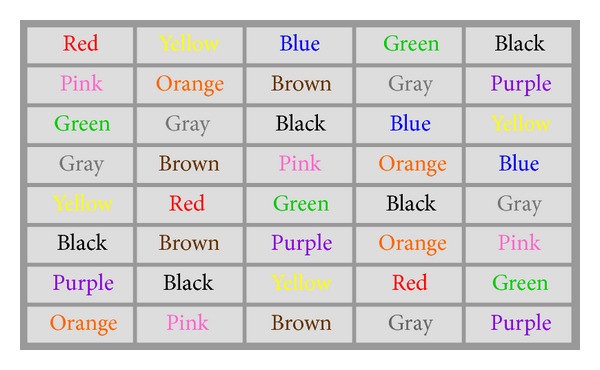
An example Stroop Test for congruent colors and words.

**Figure 2:**
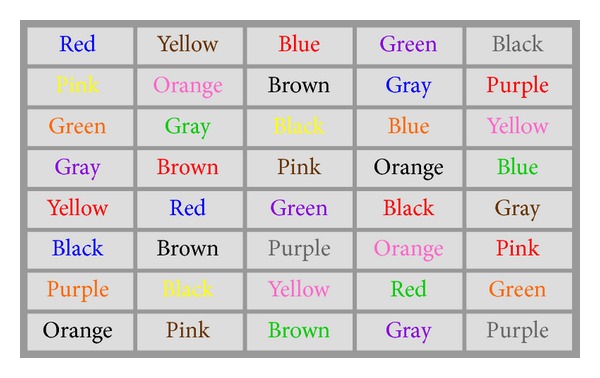
An example Stroop Test for incongruent colors and words.

Healthy human subjects (both male and female college students) were recruited in this study to perform the Stroop Test before and after physical exercise. The Stroop Test was administered on a computer screen using two sets of randomized color-recognition tests—one for congruent words and colors (Figure 1) and the other for incongruent words and colors (Figure 2). Subjects were asked to perform 20 randomized Stroop Tests before exercise and 20 randomized Stroop Tests immediately following an aerobic exercise that increases the cardiovascular activity. The timing and the errors were recorded for each Stroop Test.

The exercise was stairs climbing for 15–30 min to increase the cardiac output to approximately 50–70% of the maximal heart rate of the subject. We chose the stair-climbing exercise to increase cardiovascular output in this study for multiple reasons. It provides an objective measure of the actual total energy expenditure (and power consumption) by computing the potential energy (energy used to lift the body mass against gravity by the height traveled for the exercise duration), without using any estimation or indirect measures of energy and power. It is a purely physical exercise, without any specific mental or cognitive engagement, so that the results would not be confounded by the added cognitive variables in performing the exercise.

Heart rate, breathing rate, and partial pressure of oxygen saturation in hemoglobin were recorded before and after the exercise. In order to compare the effects of exercise versus nonexercise on executive function, the subjects were also asked to perform the same 2 sets of randomized Stroop Tests before and after nonexercise (i.e., 15–30 min of resting period) on a different day. This would eliminate any practice effects (not associated with exercise) due to the extra practices on the Stroop Test for posttests compared to the pretests. A typical experiment lasted for an hour, with 15 min of Stroop Test (pretest), 30 min of exercise (or nonexercise), and another 15 min of Stroop Test (posttest). The study protocol was approved by the Institutional Review Board.

## 3. Results

A total of sixty-five voluntary subjects were included in this study, with the average age of 22.4 ± 3.9 (mean ± standard deviation), average weight of 166.5 ± 44.7 pounds, average height of 5′8.03′′ ± 3.8′′, and average (body mass index BMI) of 24.8 ± 5.1. The average duration of exercise was 18.8 ± 6.3 min. The total height of stairs climbed was 151.4 ± 66.8 vertical meters (i.e., 496 ± 219 ft, or 34 ± 15 floors) in less than 30 min.

Note that because of the individual differences in the resting heart rate, the breathing rate, and the time spent to complete the Stroop Test, it is important to use the percentage change as a metric to compare the difference between the pre- and posttests for the same subject, so that the results are not confounded by the individual differences. In other words, the results are normalized relative to the individual subject, so that the improvements for that subject can be identified independent of the individual differences in the baseline or the resting rate.

**Figure 3:**
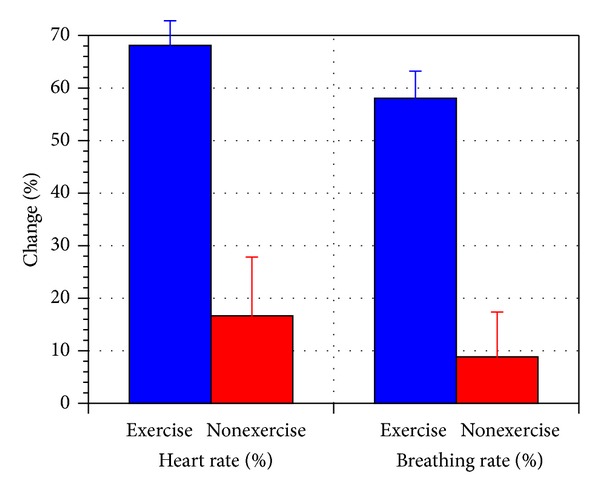
Change in heart rate and breathing rate before and after exercise (for exercise versus nonexercise trials). The error bar represents standard error of the mean.

Figure 3 shows the aerobic exercise significantly increased the heart rate by 68% and the breathing rate by 58% immediately after the exercise, compared to the nonexercise trials. The hemoglobin partial pressure of oxygen (*P*_O_2__) dropped from the average of a 96.4% saturation to a 94.6% level. This indicates that the subjects experienced a high oxygen demand during and after exercise, when the *P*_O_2__ level dropped below 96%. Normal nonhypoxic *P*_O_2__ level is 96–99%. Any level below *P*_O_2__ indicates a hypoxic condition. This occurs when the oxygen extraction exceeds oxygen delivery, resulting in a decrease in the hemodynamic *P*_O_2__ measurement.

**Figure 4:**
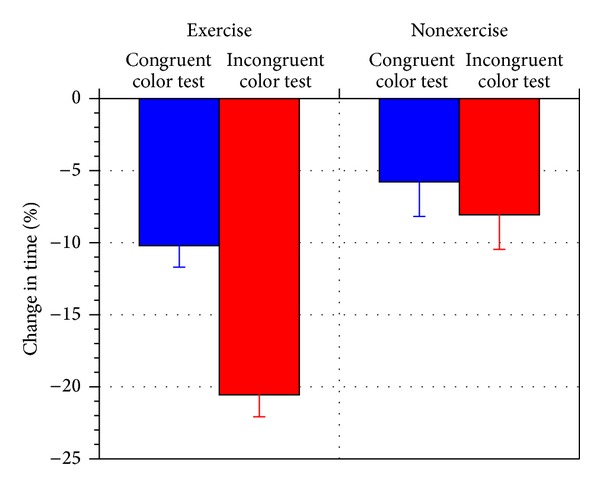
Percentage change in the time spent on completing the Stroop Tests for the congruent and incongruent color tests (for exercise versus nonexercise trials).

Figure 4 shows the percentage change in the time spent to complete the Stroop Test, before and after exercise (for exercise versus nonexercise trials). It shows that, for the congruent color test (that does not elicit conflict or interference), the time spent to complete the Stroop Test decreased by 10.2% for the exercise trials but only by 5.8% for the nonexercise trials. It shows that there is a 4.4% improvement in processing speed for the simple name-that-color test with congruent colors and words (which does not involve the attention-inhibition process).

In contrast, for the incongruent color test (which requires conflict resolution of color interference), the time spent to complete the Stroop Test decreased by 20.6% for the exercise trials but only by 8.1% for the nonexercise trials. This illustrates that exercise does produce a significant 12.5% (*P* < 0.01) improvement in processing speed for resolving color interference conflicts in executive function.

The color-recognition processing speed is doubled for the executive process that resolves color conflicts (20.6%) compared to the executive process that does not require resolve any color conflicts (10.2%). The difference in processing speed improvement between incongruent color (8.1%) and congruent color tests (5.8%) is not statistically significant for the nonexercise trials. This differentiates the specific improvements in cognitive functions induced by the increased cardiovascular activity. The processing speed improvement is doubled for a task that requires the attention-inhibition process to suppress impulsivity (incongruent color test) compared to the speed improvement for a task that does not require the attention-inhibition process (congruent color test).

**Figure 5:**
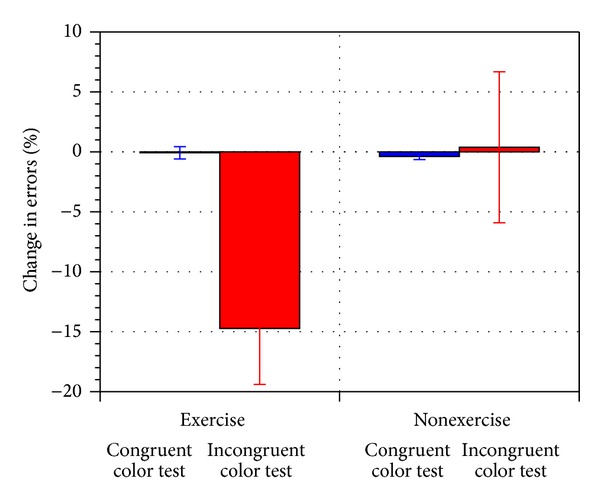
Percentage change in errors made during the Stroop Test for the congruent and incongruent color tests (for exercise versus nonexercise trials).

Figure 5 shows the percentage change in the errors made in the Stroop Tests before and after exercise (for exercise versus nonexercise trials). It shows a 15% reduction (*P* < 0.01) in the errors made for the incongruent color tests after the exercise trials, whereas there is no statistical significant change in errors made after the nonexercise trials. Note that there is little or no change in the congruent color tests because most subjects did not make any errors when the color matched the word. It is only when the color did not match the word that subjects made errors if they did not pay attention. This shows that an increase in cardiovascular activity reduced the error rate following exercise but not after sedentary resting.

## 4. Discussions

The results show that there is a significant improvement in processing executive function after less than 30 min of exercise. An increase in cardiovascular activity is demonstrated to increase the processing speed of executive function and to decrease the error rate. The improvement in processing speed is doubled for the executive process that requires the attention-inhibition process to resolve color conflict or interference compared to the executive process that does not require such conflict resolution.

As an informal observation, the subjects often reported it took a lot of mental efforts to complete the incongruent color test compared to the congruent color test. They also reported (after the exercise experiment) that they could complete the Stroop Test so much faster, as if time flied without knowing it. Most interestingly, they were surprised that the incongruent color test became so much easier without any extra mental efforts after the exercise.

This suggests that cardiovascular exercise has an immediate effect on improving the executive function for both congruent and incongruent color Stroop Tests. The improvement in processing speed is doubled for the highly demanding task that requires resolving color conflict or interference (incongruent color test) compared to the less demanding task that does not require resolution of color conflict (the congruent color test). That is, aerobic exercise can improve performance by suppressing the impulsivity that interferes with conflict resolution, immediately following an increase in cardiovascular activity. It improves the performance by increasing the processing speed and by reducing the errors made in resolving the color conflict in the Stroop Test.

These findings are consistent with those of other studies that reported aerobic exercise accelerated reaction time compared with the rest condition. The improvement is specific to Stroop Test that requires conflict resolution in both acute aerobic and strength exercises but not in the Trail Making Test (for testing visual attention and task switching, which does not require conflict resolution skills) [24]. Our results are also consistent with the findings that the improvement is related to cardiovascular activities but not cognitive engagement. Using exergaming as the physical activity, other studies have shown that exercise enhanced children's speed to resolve interference from conflicting visuospatial stimuli, but cognitive engagement (of the exercise game) had no effect on any aspect of task performance [25]. In order to further identify the neural circuitry involved in the executive function, Rueda et al. [26] had developed an integrated Attention Network Test (ANT) using flanker task [27] to measure the efficiency of three networks in adults. It showed that moderate aerobic exercise modulated the functioning of phasic alertness by increasing the general state of tonic vigilance [28], but aerobic exercise did not modulate the functioning of either the orienting or the executive control attentional networks. This may account for the dissimilar results based on other executive function tests, because these tests tap into the selective attention and orientation responses rather than into the conflict resolution process for interference. Taken together, these results show that aerobic exercise can increase the processing speed in resolving conflicting interference and reduce the error rates that are related to the cardiovascular activity, without necessarily being confounded by any mental cognitive engagement while doing such exercise. This improvement in executive function may be specific for resolving conflicts of interference only but not necessary for improving visual attention or task switching processes.

Note that this is one of the first experiments that identified the specific cognitive improvements attributable to the immediate effects following an increase in cardiovascular activities. As the first phase in the experimental design, the present study included a wide range of healthy subjects (independent of the physical fitness level or mental capacity). The present sample in this study included a wide range of BMI, with an average of 24.8 ± 5.1. This suggests that the cognitive improvements were found in both physically fit and obese subjects. Given that we have demonstrated the acute effects of cardiovascular activities on cognitive functions, the next phase in our experimental design will compare the specific cognitive improvements between physically active and sedentary subjects and between healthy and attention deficit hyperactivity disorder (ADHD) subjects. Since the Stroop Test is often used to identify the impulsivity of the subjects, comparing the difference between ADHD and healthy subjects will provide specific evidence identifying whether physical exercise will make ADHD subjects become more impulsive (an increase in both processing speed and error rate) or pay more concentrated attention (an increase in processing speed but a reduction in error rate). Future experiments will identify whether sedentary subjects will benefit more than physically fit subjects or ADHD subjects will benefit more than normal subjects.

## 5. Summary

This study has demonstrated that an increase in cardiovascular activity could have immediate effects on increasing the processing speed and reducing the errors in executive control. It doubles the executive function processing speed of the highly demanding task to resolve conflict or interference compared to the less demanding task that does not require resolving color conflicts. It suggests that the improvement is more specific to enhancing the attention-inhibition process than to the executive process that does not require such attention-inhibition. The improvement is observed immediately after less than 30 min of aerobic exercise. Little or no improvement is observed for the nonexercise control group after similar period of sedentary resting.
